# Toll-like receptor 4 confers inflammatory response to Suilysin

**DOI:** 10.3389/fmicb.2015.00644

**Published:** 2015-06-26

**Authors:** Lili Bi, Yaya Pian, Shaolong Chen, Zhiqiang Ren, Peng Liu, Qingyu Lv, Yuling Zheng, Shengwei Zhang, Huaijie Hao, Yuan Yuan, Yongqiang Jiang

**Affiliations:** ^1^State Key Laboratory of Pathogen and Biosecurity, Beijing Institute of Microbiology and EpidemiologyBeijing, China; ^2^Beijing Key Laboratory of Immunology Regulatory and Organ Transplantation, Basic Research Lab of Organ Transplant Institute, 309th Hospital of the People's Liberation ArmyBeijing, China; ^3^CAS Key Laboratory of Pathogenic Microbiology and Immunology, Institute of Microbiology, Chinese Academy of ScienceBeijing, China

**Keywords:** SLY, TLR4, MAPK, inflammation

## Abstract

*Streptococcus suis* serotype 2 (SS2) is an emerging human pathogen worldwide. A large outbreak occurred in the summer of 2005 in China. Serum samples from this outbreak revealed that levels of the main proinflammatory cytokines were significantly higher in patients with streptococcal toxic-shock-like syndrome (STSLS) than in patients with meningitis only. However, the mechanism underlying the cytokine storm in STSLS caused by SS2 remained unclear. In this study, we found that suilysin (SLY) is the main protein inflammatory stimulus of SS2 and that native SLY (nSLY) stimulated cytokines independently of its haemolytic ability. Interestingly, a small amount of SLY (Å Mol/L) induced strong, long-term TNF-α release from human PBMCs. We also found that nSLY stimulated TNF-α in wild-type macrophages but not in macrophages from mice that carried a spontaneous mutation in TLR4 (P712H). We demonstrated for the first time that SLY stimulates immune cells through TLR4. In addition, the Myd88 adaptor-p38-MAPK pathway was involved in this process. The present study suggested that the TLR4-dependent inflammatory responses induced by SLY in host might contribute to the STSLS caused by SS2 and that p38-MAPK could be used as a target to control the release of excess TNF-α induced by SS2.

## Introduction

The Gram-positive bacterium *Strptococcus suis* serotype 2 (SS2) can cause a systemic infection in humans; sepsis and meningitis are the most common clinical manifestations (Tang et al., 2006; Wangkaew et al., 2006; Huong et al., 2014). Severe sepsis can lead to streptococcal toxic-shock-like syndrome (STSLS). In 2005, China reported more than 200 human cases with an unusual clinical presentation of STSLS and a mortality rate of up to 20% (Sriskandan and Slater, 2006). Serum samples revealed that TNF-α, IL-6,IL-1β, IL-8, IL-12p70 and IFN-γ were significantly higher in patients with STSLS than in those with meningitis (Ye et al., 2009).

It was reported that cell walls and SLY can activate endothelial cells to release cytokines (Vadeboncoeur et al., 2003). Stimulation of human monocytes with whole encapsulated *S. suis* or purified cell wall components influences the expression of TLR2 and CD14 mRNA (Graveline et al., 2007). Recombinant SLY (rSLY) triggers the production of TNF-α by human monocytes and IL-6 by pig PAMs and monocytes (Lun et al., 2003). SLY is a 497 amino-acid protein belonging to the cholesterol-dependent cytolysin (CDC) family, which has more than 20 members, including perfringolysin O and streptolysin O, expressed by *Clostridium perfringens* and *Streptococcus pyogenes*, respectively. Like other members of the CDC family produced by Gram-positive bacteria, a classical feature of these toxins is their ability to create transmembrane pores in cholesterol-containing membranes thereby causing cell lysis (Jacobs et al., 1994; Gottschalk et al., 1995).

Sequence type 7 (ST-7) strains caused the human outbreak in China in 2005 and were more toxic to human peripheral blood mononuclear cells (PBMCs) than ST-1 strains (mainly referring to the European virulent strains) (Ye et al., 2006). Interestingly, the ST-7 strains produced more SLY than the non-epidemic strains, and this contributed to invasive infection (He et al., 2014). However, the mechanisms underlying the SLY induced inflammatory responses of the host have not been fully elucidated.

The pattern-recognition receptors (PRRs) in the host play important roles in recognizing bacterial pathogens (Akira et al., 2006). The PRRs, including Toll-like receptors (TLRs), RIG-I-like receptors, NOD-like receptors and C-type lectin receptors, recognize distinct microbial components to activate immune cells (Takeuchi and Akira, 2010). TLRs are expressed in various immune cells, including lymphocytes, monocytes and dendritic cells (DCs) in blood and macrophages in tissue; and even in non-immune cells, including fibroblasts, endothelial, and epithelial cells. TLR4 usually was reported to recognizes the lipopolysaccharides (LPS) from gram-negative bacteria (Akira and Takeda, 2004; Kawai and Akira, 2006; Lu et al., 2008). TLR4 can also confer responsiveness to the pneumolysin from *Streptococcus pneumonia*e (Malley et al., 2003).

In this study, we eliminated the influence of LPS and found that nSLY activates several types of immune cells via TLR4, including human PBMCs from human blood, primary peritoneal macrophages from mice, THP-1 and RAW264.7 macrophages. Moreover, this inflammatory activity is independent of its haemolytic ability. Interestingly, we also found that the TLR4-MyD88-p38 MAPK signal transduction pathway is essential for SLY-induced inflammatory cytokine synthesis.

## Materials and methods

### Reagents

Cell culture media, fetal bovine serum (FBS), penicillin G, streptomycin, and β-mercaptoethanol were purchased from GIBCO. Gel-filtration chromatography -purified Escherichia coli O127:B8 LPS was purchased from Sigma. Antibodies (Abs) for total and phospho-specific MAPKs were purchased from Cell Signaling Technology. The MAPK inhibitors: SB203580 (p38) and SB202474 (the negative control for SB203580), U0126 (ERK), and U0124 (the negative control for U0126) and SP600125 (JNK) were purchased from Calbiochem. Polymyxin B, CLI-095, Pepinh-Control, Pepinh-MYD, Pepinh-TRIF, and Lipofectamine2000 were purchased from Invitrogen. Dual Luciferse Reporter Assay Kits and Cyto Tox 96 Non-Radioactive Cytotoxicity Assay Kits were purchased from Promega.

### Bacterial strains

The virulent SS2 strain 05ZYH33 that belongs to sequence type 7 (ST-7) based on multilocus sequence typing, was originally isolated from a STSLS patient in China in 2005 (Ye et al., 2009). ΔSLY, ΔMRP, ΔFhb, Δ1083, and Δ1881 were the isogenic mutants of *sly*, *mrp*, *fhb*, the *ABC transporter 1083* and *HP1881* from 05ZYH33 (Pian et al., 2015), respectively, that were constructed in our laboratory. The bacteria were grown overnight on goat blood agar at 37°C, and isolated colonies were inoculated into Todd–Hewitt broth (THB, Difco).

### Ethics statement

The healthy donors who provided serum and plasma for this study at the 307 Hospital provided written informed consent in accordance with the Declaration of Helsinki. Approval was obtained from the medical ethics committee of the 307 Hospital. This research was approved by the ethics committee on Animal Experimentation of the Chinese Association for the Accreditation of Laboratory Animal Care (CAALAC) and the relevant local animal welfare bodies in China. In addition, the permit number for all of the animal work was SCXK-(JUN)2013-029), and this work was approved by the animal ethics committee of the Beijing Institute of Microbiology and Epidemiology. All efforts were made to minimize the suffering of the animals employed in this study.

### Real-time PCR analysis

The gene expression of proinflammatory cytokines (IL-1β, IL-6, and TNF-α) in hCMEC/D3 cells, induced by *S. suis* strains (MOI = 10:1, stimulated for 2 h), was calculated as the mean of the fold increase of in mRNA above that of the PBS group as determined by real-time PCR analysis. Gene-specific primers (Listed in Table S1) were designed to produce an amplicon of 100–150 bp for each cytokine gene tested. The contaminating DNA in the samples was removed by using Amibion's DNA-free Kit (Applied Biosystems, Foster City, CA). cDNA was generated by random hexamer primers, and real-time RT-PCR was performed in triplicate using the Step One Plus system together with the SYBR Green master mix. The relative mRNA level were determined by calculating the threshold cycle (ΔCt) of each gene using the classic ΔCt method. Negative controls were performed by using cDNA generated without reverse transcriptase as templates. were also included as blank controls. The β-actin gene was used as an internal control to normalize all the other genes. The samples from at least three independent experiments were analyzed.

### The inflammatory activity induced by SLY *in vivo*

Six-weeks-old female ICR mice were randomly divided into three groups with six mice per group. The three groups of mice were challenged intraperitoneally (i.p.) with 0.3 ml (~5 × 10^8^ CFU/ml) of stationary-phase WT 05ZYH33, the *sly* mutant ΔSLY or THB. At certain times post-infection (4, 8, and 12 h), mice in each group were euthanized. The sera isolated from the blood were used to determine IL-1β, IL-6, TNF-α, and IL-10 levels with the Mouse Cytokine 10-Plex Panel (Merck).

### Purification of SLY protein and removal of endotoxins

The purification and characterization of native SLY (nSLY) were performed as previous report (Jacobs et al., 1994; Lv et al., 2011). Recombinant expressed SLY (rSLY) and the SLY^P353V^ mutant proteins used in this study were previously reported (Ren et al., 2012). Endotoxins were removed with Triton X-114 (Liu et al., 1997). The remaining endotoxin contamination in proteins, cell culture medium and solutions contained less than 0.03 EU/ml, a concentration below that which is known to cause cell activation (Martin and Dorf, 1991).

### Titration of haemolytic activity

The nSLY, rSLY, and SLY^P353V^ proteins were evaluated via titration of their haemolytic activity as in our previous study (Hao et al., 2013). Briefly, serial two-fold dilutions of the proteins were prepared in polystyrene deep-well titer plates with PBS (with or without 0.1% β-mercaptoethanol). Subsequently, an equal amount of human red blood cells (RBC) (final concentration of RBS: 2%) was added to each well. Following incubation for 1 h at 37°C, the mixtures were centrifuged (1500 g for 10 min), the supernatants were transferred to polystyrene microplates, and their absorbance was measured at 540 nm with a microELISA reader. Triton X-100 was used as the positive control. Haemolytic units (HU) were calculated from the endpoint (defined as 50% lysis). The endpoint was estimated as the well in which the erythrocyte pellet was half the size of those in control wells without added proteins.

### Cell lines and isolation of primary cells

THP-1 cells, a monocytic cell line that treated with PMA (50 ng/ml) for 48 h to induce macrophage differentiation. RAW264.7, a macrophage cell line, were maintained in RPMI 1640 supplemented with 10% (v/v) FBS. Human embryonic kidney 293T cells (HEK) were maintained in DMEM supplemented with 10% (v/v) FBS. The hCMEC/D3, an immortalized human cerebral microvascular endothelial cell line, was maintained in EBM-2 basal medium with fetal bovine serum (5%), penicillin-streptomycin (1%), hydrocortisone (1.4 μM), ascorbic acid (5 μg/ml), chemically defined lipid concentrate (1/100), HEPES (10 mM) and bFGF (1 ng/ml). Primary peritoneal macrophages were isolated from mice as described (Malley et al., 2003). Human PBMCs were prepared from fresh heparinized blood from healthy volunteers. The blood was diluted 1/1 in PBS and layered on top of Ficoll-Paque Plus (Amersham Biosciences). After centrifugation at 1000 g for 20 min at room temperature, the PBMC layer was collected and washed twice in medium. All cell culture experiments were conducted under sterile conditions, and cells were cultured at 37°C in a 5% CO_2_ atmosphere.

### The cytotoxicity of nSLY to immune cells

The immune cells (PBMCs, primary peritoneal macrophages from mice, THP-1 and RAW264.7 macrophages) were added at a density of 1 × 10^6^ cells/ml into 96-well culture plates. The nSLY was added to the cell culture wells to a concentration of 100 ng/ml. The cytotoxicity of nSLY to immune cells was detected with Cyto Tox 96 Non-Radioactive Cytotoxicity Assay Kits after 4 h of incubation.

### Cytokine detection by enzyme-linked immunosorbent assay (ELISA)

PBMCs, THP-1 macrophages, RAW264.7 macrophages and primary macrophages from mice (1 × 10^6^ cells/ml in RPMI 1640 supplemented with 10% FBS) were stimulated with protein or LPS for 16 h at 37°C in a 5% CO_2_-95% air incubator. Cells were pelleted by centrifugation, and the cytokine concentrations in the supernatants were determined by ELISA according to the manufacturer's protocol (R&D Systems).

### Western blotting

Proteins were separated on SDS-polyacrylamide gels and then transferred to polyvinylidene difluoride (PVDF) membranes. The membranes were blocked with 5% (w/v) BSA for 1 h at room temperature and then incubated overnight at 4°C with primary antibodies in TBST (0.1%, v/v) with 1% (w/v) BSA. After washing, the membranes were incubated with HRP-conjugated secondary antibodies (Cell Signaling Technology) for 1 h at room temperature, followed by development with a BM chemiluminescence reagent (Roche).

### NF-κB luciferase reporter gene assays

HEK293T and RAW264.7 cells were transiently transfected with Lipofectamine 2000 transfection reagent (Invitrogen) according to the manufacturer's instructions. After 24 h, the cells were stimulated with LPS (100 ng/ml) or SLY (100–800 ng/ml) for 6 h before lysis.

### Statistical analysis

ELISA results are expressed as the mean ± SD of the pg/ml values. Differences between two groups were analyzed using the unpaired two-tailed Student's *t*-test; if Levene's test was not significant (*P* > 0.05), a Mann-Whitney tests were used. A One-Way analysis of variance (ANOVA) was used to compare the means of three or more samples. For all tests, a value of *P* < 0.05 was considered as the threshold for significance. All statistical analyses were carried out using SPSS 15.0 (SPSS Inc., Chicago, IL, USA).

## Results

### SLY is the main *S. suis* protein that stimulates inflammation

**Figure 1:**
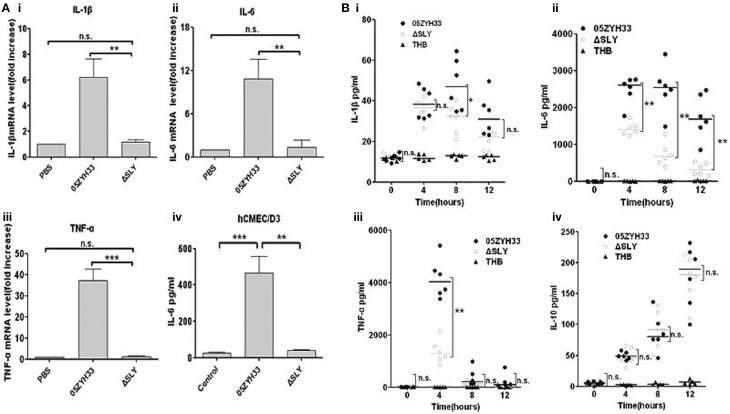
**SLY is the main protein in** ***S. suis*** **that stimulates inflammation**. (**Ai-iii**) The gene expression of proinflammtory cytokines (IL-1β, IL-6 and TNF-α) in hCMEC/D3 cells induced by *S. suis* strains (MOI = 10:1, stimulated for 2 h). Data are expressed as the mean ± SD of the fold increase of mRNA above the PBS group for at least three independent experiments. (**Aiv**) The release of interleukin-6 (IL-6) by hCMEC/D3 was detected after stimulation with a live *S. suis* strain 05ZYH33 or its ΔSLY mutant (MOI = 10:1) for 3 h. Data are expressed as mean ± SD for at least three experiments and are analyzed by Student's unpaired *t*-test. **(B)** The levels of cytokines in ICR mouse serum after i.p. infection with 0.3 ml of live *S. suis* culture liquid (10^8^ CFU/ml). Each symbol represents the serum cytokine levels (pg/ml) in one mouse. Horizontal lines indicate the median for each group, and the data were analyzed by the Mann-Whitney test. n.s. no significance. ^*^P < 0.05; ^**^P < 0.01; ^***^P < 0.001. n.s. no significance; ΔSLY, an isogenic mutant of *sly* from 05ZYH33; THB, Todd-Hewitt broth.

To identify the inflammatory stimulus of *S. suis*, several genes encoding potential virulence factors, including SLY (12), MRP (Vecht et al., 1996; Yang et al., 2006; Rehm et al., 2007), Fhb (Pian et al., 2012), HP1083 and HP1881 were knocked out in our laboratory, and then the mutant and WT strains were compared for their inflammatory capacities. Surprisingly, in hCMEC/D3 cells, only ΔSLY exhibited a significant decrease in its capacity to induce the transcription of proinflammatory cytokines (TNF-α, IL-6, IL-1β, and IL-8) compared with the WT strain (Figure 1A and Figure S1). The obvious loss of IL-6 release from hCMEC/D3 induced by ΔSLY, compared to the WT, was verified by ELISA (Figure 1B). To evaluate the roles of SLY-induced inflammation *in vivo*, 4-week-old SPF ICR mice (six mice/group) were challenged i.p. with WT 05ZYH33 and the mutant ΔSLY; and then the serum levels of cytokines were analyzed at different times. The serum levels of the proinflammatory cytokines (TNF-α, IL-6 and IL-1β) were significantly higher in mice infected with WT 05ZYH33 than in those infected with ΔSLY at specific time point (Figure 1Bi–iii). However, low levels of the anti-inflammatory cytokine IL-10 were detected with no difference between the WT and the mutant ΔSLY (Figure 1Biv). These results indicated that SLY was an important inflammatory stimulus of *S. suis*.

### SLY is a strong inflammatory activator of PBMCs

**Figure 2:**
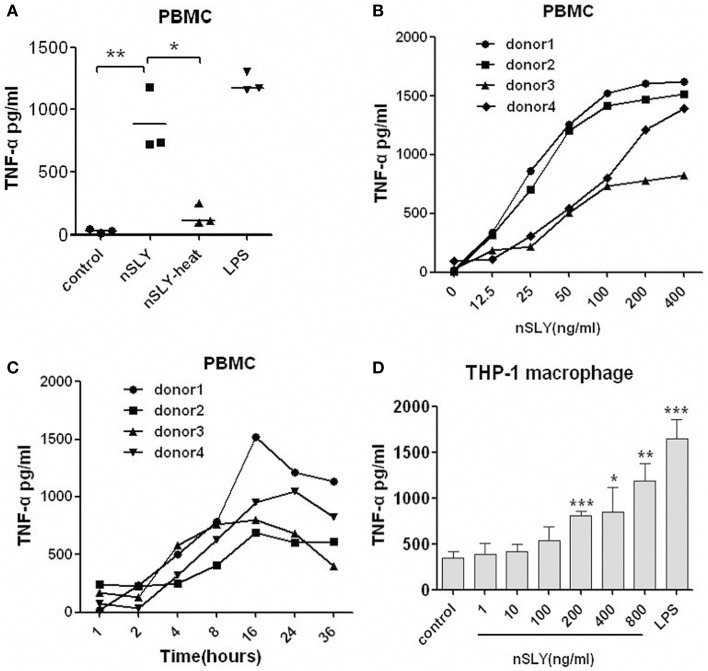
**SLY induces strong, long-term TNF-α release from human PBMCs**. **(A)** Heated, inactivated nSLY could not induce TNF-α release from PBMCs. The nSLY was heated at 100°C for 30 min and then incubated with PBMCs at a concentration of 100 ng/ml. Culture supernatants were harvested and assayed for TNF-α production by ELISA. Each symbol represents the level of TNF-α (pg/ml) released from the PBMCs from one donor in one independent experiment. Horizontal lines indicate the mean for each group, and the data in were analyzed by Student's unpaired *t*-test. Dose **(B)** and time-course **(C)** of production of TNF-α in PBMCs from different donors treated with nSLY (100 ng/ml). Dose response **(D)** of TNF-α production in THP-1 macrophage cells treated with nSLY or LPS. Culture supernatants were harvested and assayed for cytokine production by ELISA. Data are expressed as the mean ± SD for three independent experiments. nSLY, native SLY; nSLY-heat, the native SLY was heated at 100°C for 30 min. ^*^P < 0.05; ^**^P < 0.01; ^***^P < 0.001. The comparisons between test groups and the control in **(D)** were analyzed by a One-Way ANOVA test.

Unlike most other cytokines, TNF-α administered *in vivo* produces dramatic physiological changes and large doses of TNF-α can result in tissue damage and metabolic changes that clearly mimic endotoxic shock (Tracey et al., 1987; Hesse et al., 1988; Whicher and Evans, 1990). TNF-α was chosen as the main marker of inflammatory activity in the present study. We purified the nSLY from the supernatant of a bacterial culture as in our previous study (Ren et al., 2012) and detected its ability to induce the TNF-α release from immune cells. To exclude the possibility that the observed inflammatory activity of nSLY is attributable to contaminating LPS or other endotoxins, removal of endotoxin from the proteins was carried out. Subsequently, the final endotoxin contamination for nSLY, rSLY, and SLY^P353V^ used in this study is less than 0.03 EU/ml, a concentration below that which is known to cause cell activation (Martin and Dorf, 1991). Moreover, we used heat to abolish the activity of nSLY to induce TNF-α release from PBMCs. As shown in Figure 2A, the inflammatory response was lost when nSLY was heated (at 100°C for 30 min), indicating that this activity of nSLY was due to the protein, not to endotoxin contamination.

We also found that nSLY induces TNF-α release from PBMCs (Figure 2B), THP-1 macrophages (Figure 2D), RAW264.7 cells (**Figure 6C**) and primary peritoneal macrophages from C57BL/6 (**Figure 4C**) and C3H/HeN mice (**Figure 4D**). A strong activator of PBMC, 100 ng/ml nSLY induced the higher TNF-α release from PBMCs than from other macrophages (Figures 2B,D, **4C,D**, **6C**). Then we performed serial concentrations of nSLY with different incubation times with the PBMCs. Interestingly, as shown in Figure 2B, PBMCs were more sensitive and demonstrated a stronger response to SLY than THP-1 macrophages (Figures 2B,D). Furthermore, unlike the mouse infection model (Figure 1B), SLY produced long-term stimulation of TNF-α release from PBMCs isolated from human blood (Figure 2C). Therefore, *in vitro* assays with cells following nSLY stimulation for 16 h were performed.

### The inflammatory activity of nSLY is independent of its haemolytic activity

**Figure 3:**
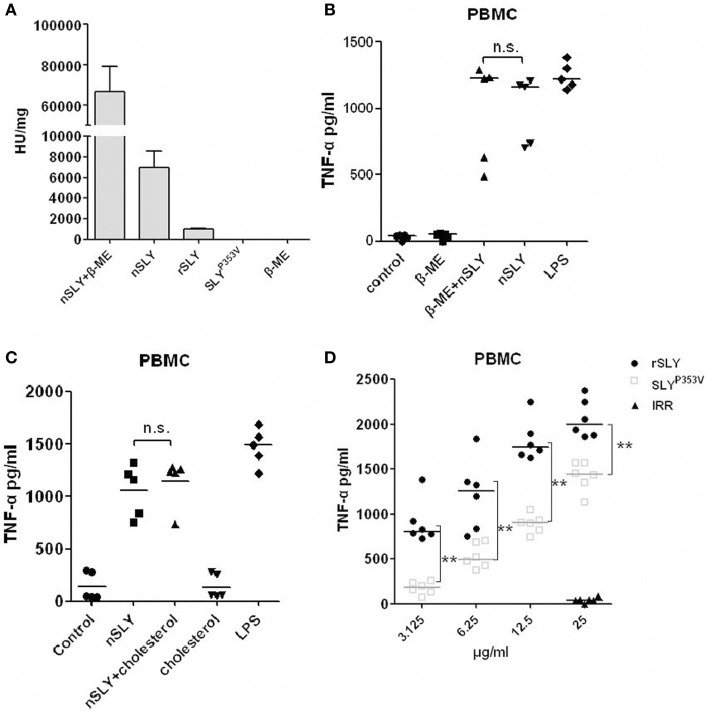
**The inflammatory activity of nSLY is independent of its haemolytic activity. (A)** The haemolytic activities of nSLY, rSLY, and SLY^P353V^ were tested. One haemolytic unit is defined as the reciprocal of the SLY titre, which was calculated as the highest dilution of the protein that caused at least 50% haemolysis. The data are expressed as the mean ± SD for three independent experiments. **(B,C)** The nSLY induced-TNF-αrelease is independent of its haemolytic activity. **(B)** PBMCs were isolated from healthy volunteers and incubated with nSLY (100 ng/ml) pretreated with 0.1% β-ME or with nSLY (100 ng/ml; no pretreatment), LPS (100 ng/ml) was used as a positive control. The concentration of TNF-α was measured by ELISA. Each symbol represents the level of TNF-α (pg/ml) released from the PBMC from one donor in one independent experiment. Horizontal lines indicate the median for each group, and the data were analyzed by the Mann-Whitney test. n.s. no significance. **(C)** PBMCs were isolated from healthy volunteers and incubated with nSLY (100 ng/ml) pretreated with cholesterol (1.5 μg/ml) or with nSLY (100 ng/ml; no pretreatment), LPS (100 ng/ml) was used as a positive control. The concentration of TNF-α was measured by ELISA. Each symbol represents the level of TNF-α (pg/ml) released from the PBMC from one donor in one independent experiment. Horizontal lines indicate the median for each group, and the data were analyzed by the Mann-Whitney test. n.s. no significance. **(D)** The rSLY induced TNF-αrelease is affected by its haemolytic activity. PBMCs were incubated with SLY^P353V^ (3.125–25 μg/ml), rSLY (3.125–25 μg/ml) or an irrelevant protein (25 μg/ml) for 16 h. The concentration of TNF-α was measured by ELISA. The rSLY, SLY^P353V^ and irrelevant protein (IRR) were purified by the same procedure. Each symbol represents the levels of TNF-α (pg/ml) released from the PBMC from one donor in one independent experiment. Horizontal lines indicate the median for each group, and the data were analyzed by the Mann-Whitney test. ^**^P < 0.01.

Cellular necrosis or cleavage can induce inflammatory reactions; therefore, the inflammatory activity of SLY could depend on its haemolytic activity. Members of the CDC family share similar functions: they bind to the target cell membrane via interactions with specific receptors, including cholesterol and CD59 (Giddings et al., 2004; Xu et al., 2010). Cholesterol can obviously inhibit their haemolytic ability. To determine whether the role of SLY in stimulating cytokines production requires its haemolytic activity, we purified recombinant expressed SLY (rSLY) and the SLY variant containing a point mutation (SLY^P353V^) which lost all hemolytic activity (Ren et al., 2012). We found that nSLY without β-ME had less haemolytic activity (5079 U/mg) than nSLY supplemented with β-ME (81267 U/mg). rSLY had even less haemolytic activity (1026 U/mg), and SLY^P353V^ lost all haemolytic activity (Figure 3A), indicating that the purified nSLY and rSLY lost most of their haemolytic activity without β-ME to reduce the disulphide bond. The concentration of 100 ng/ml (without haemolytic activity and cytotoxicity to PBMCs, data not shown) of nSLY is sufficient to induce a strong response in PBMCs and the release of TNF-α (Figure 3B). Additionally, β-ME had a limited effect on nSLY-induced TNF-α release (Figure 3B). Moreover, we found that cholesterol had no effect on the inflammatory ability of nSLY in PBMCs (Figure 3C and Figure S2). Although SLY^P353V^ induced less TNF-α release than rSLY at the same concentration in PBMCs, SLY^P353V^ preserved its inflammatory activity (Figure 3D). These results confirmed that the inflammatory activity of nSLY is different from that of rSLY, and that it is independent of its haemolytic activity, suggesting that the inflammatory activity of nSLY might depend on a specific host receptor, but not on cellular necrosis or cleavage due to the cytotoxicity of SLY.

### The inflammatory activity of SLY occurs in a TLR4-dependent manner

**Figure 4:**
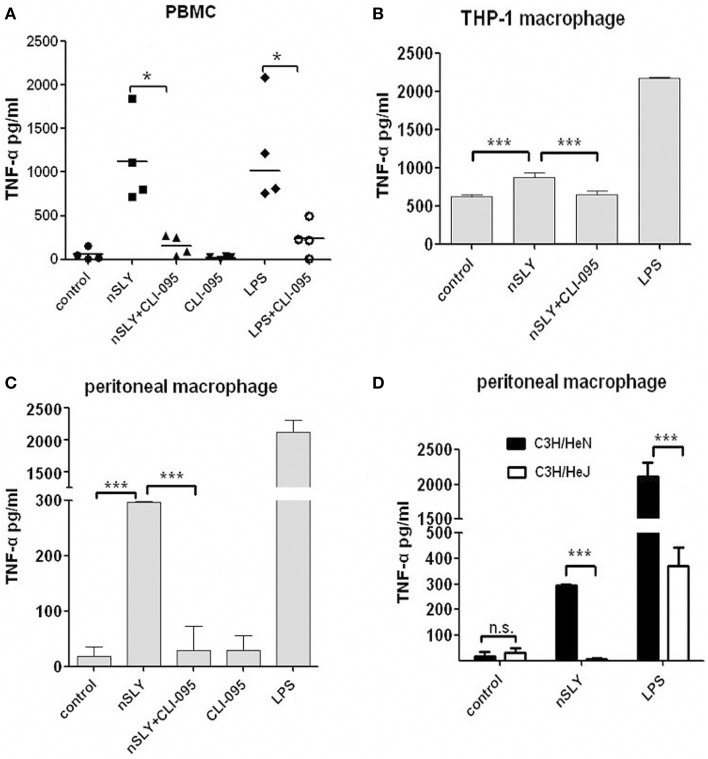
**TLR4 on immune cells mediated the inflammatory activity of SLY**. PBMCs **(A)**, THP-1 macrophages **(B)** or primary peritoneal macrophage isolated from C57BL/6 mice. **(C)** were pre-incubated with CLI-095 (6.4 μM) for 6 h, and then nSLY (100 ng/mL) or LPS (100 ng/ml) was added for another incubation for 16 h. In **(A–C)**, the culture supernatant were harvested and assayed for cytokine production by ELISA. Each symbol in **(A)** represents the level of TNF-α (pg/ml) released from PBMC from one donor in one independent experiment. Horizontal lines indicate the median for each group, and the data were analyzed by the Mann-Whitney test. **(D)** Primary peritoneal macrophages isolated from C3H/HeN (black) or C3H/HeJ (white) mice were incubated with LPS (100 ng/mL) or nSLY (100 ng/mL) for 16 h. Concentrations of TNF-α in sera from mice were measured by ELISA. The data in **(B–D)** are expressed as the mean ± SD for three independent experiments. The data were analyzed by Student's unpaired *t*-test. ^*^P < 0.05; ^***^P < 0.001; n.s., no significance; C3H/HeN, the WT mice; C3H/HeJ, the mice carry a missense point mutation in the TLR4 gene (P712H).

Pneumolysin (PLY) of *S. pneumoniae*, induces a TLR4-dependent proinflammatory response in macrophages (Malley et al., 2003). The mechanisms underlying SLY induced inflammation was studied in this study. First, we used CLI-095, a specific inhibitor of TLR4 (Ii et al., 2006; Kawamoto et al., 2008) and found that it blocked the SLY-induced cytokine release in PBMCs (Figure 4A), THP-1MΦ cells (Figure 4B) and peritoneal macrophages from C57BL/6 mice (Figure 4C). Second, we compared TLR4 dependent SLY inflammatory activity in macrophages isolated from wild-type mice (C3H/HeN) with activity in macrophages isolated from TLR4 point-mutant mice (C3H/HeJ). C3H/HeJ mice carry a missense mutation in the TLR4 gene (P712H), which renders them hypo-responsive to LPS (Poltorak et al., 1998). As shown in Figure 4D, SLY activates the synthesis of TNF-α in peritoneal-derived macrophages from WT mice but could not activate mutant peritoneal-derived macrophages, demonstrating that SLY induces inflammatory cytokine production through TLR4 on immune cells.

### MyD88 is the main adaptor protein in the SLY-TLR4 signaling pathway

**Figure 5:**
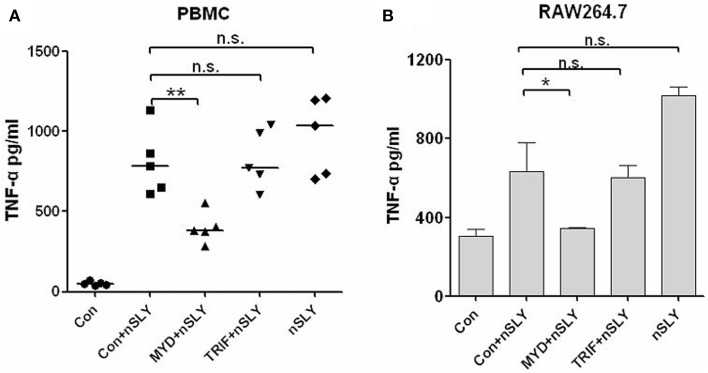
**SLY-activated immune cells produce TNF-α primarily through the MyD88 adaptor**. PBMCs **(A)** or RAW264.7 **(B)** were pretreated with 10 μM of pepinh-control (con), pepinh-MYD (MYD), or pepinh-TRIF (TRIF) for 6 h and then they were incubated with LPS (100 ng/ml) or SLY (100 ng/ml) for 16 h. The concentrations of TNF-α were measured in the supernatants by ELISA. Each symbol in **(A)** represents the level of TNF-α (pg/ml) released from PBMCs from one donor in one independent experiment. Horizontal lines indicate the median for each group, and the data were analyzed by the Mann-Whitney test. The data in **(B)** are expressed as the mean ± SD for three independent experiments. The data were analyzed by Student's unpaired *t*-test. ^*^P < 0.05; ^**^P < 0.01; n.s., no significance; Con, pepinh-control; pepinh-MYD, the inhibitor of MyD88; pepinh-TRIF, the inhibitor of TRIF.

TLR4 triggers MyD88-dependent and TRIF-dependent signaling (Lu et al., 2008). MyD88 is required for full signaling in the TLR pathway. To discriminate MyD88- or TRIF-mediated signaling downstream of SLY-TLR4, we used MyD88 and TRIF- specific inhibitors (pepinh-MYD and pepinh-TRIF, respectively) and pepinh-Control in this study. Pepinh-MYD inhibited SLY-induced TNF-α release in PBMCs (Figure 5A) and RAW264.7 (Figure 5B). However, pepinh-TRIF played only a limited role for PBMCs and RAW264.7 (with no significant effects compared with the pepinh-Control treatment), indicating that MyD88 is the main downstream adaptor of TLR4 in SLY-induced TNF-α synthesis (Figure 5B).

### Involvement of MAPK in TNF-α release by immune cells stimulated with SLY

**Figure 6:**
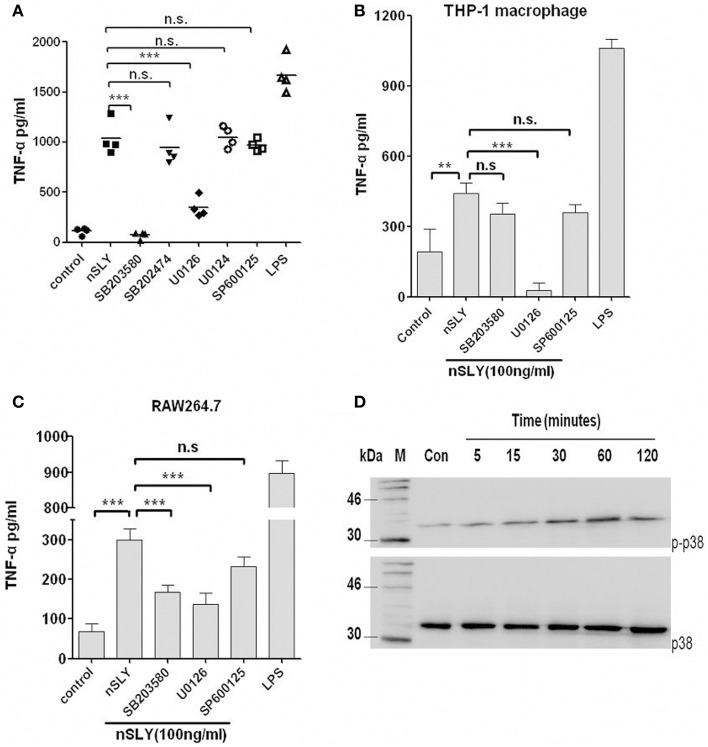
**MAPK is involved in SLY-induced TNF-α release by immune cells**. PBMCs **(A)**, THP-1 macrophages **(B)** or RAW264.7 macrophages **(C)** were pretreated with 10 μM of SB203680,SB202474, U0126, U0124, or SP600125 for 1 h, and then they were incubated with LPS (100 ng/ml) or SLY (100 ng/ml) for 16 h. Culture supernatants were harvested and assayed for cytokine production by ELISA. Each symbol in **(A)** represents the level of TNF-α (pg/ml) released from PBMCs from one donor in one independent experiment. Horizontal lines indicate the mean for each group. Data in **(B,C)** are expressed as the mean ± SD for three independent experiments. All data were analyzed by Student's unpaired *t*-test. ^**^P < 0.01; ^***^P < 0.001; n.s., no significance. MAPK inhibitor, SB203580 (p38), and SB202474 (the negative control for SB203580), U0126 (ERK), and U0124 (the negative control for U0126), SP600125 (JNK). **(D)** The p38-MAPK phosphorylation induced by nSLY. PBMCs were incubated with nSLY (100 ng/ml) for different times (min) and then lysed for SDS-PAGE and Western blot. The total/phosphorylated p38 was detected by Abs against human total/phospho-specific p38 and HRP-conjugated secondary antibodies.

Mitogen-activated protein kinase (MAPK) is an important serine/threonine protein kinase in inflammatory development. Four MAPK pathways are activated upon LPS-TLR4 stimulation in macrophages, including ERK, JNK/SAPK, p38, and BMK/ERK5. We analyzed whether these MAPKs are involved in SLY induced inflammatory response. We used inhibitors of p38 (SB203580), ERK (U0126) and JNK (SP600125) and found that SB203580 had a powerful effect on PBMCs (Figure 6A), but limited effect on RAW264.7 (Figure 6C) and it had no effect on THP-1 macrophages (Figure 6B). In contrast, U0126 inhibited inflammatory activity in all cell types tested (Figures 6A–C), but SP600125 had no effect on these cell types. MAPK may play a co-operative role in TNF-α synthesis when SLY activates immune cells. We then used western blotting to evaluate the activation of MAPKs. ERK-MAPK phosphorylation was not detected in PBMCs and RAW264.7 (data not shown). As shown in Figure 6D, nSLY induced p38-MAPK phosphorylation in PBMCs within 2 h, indicating that SLY can activate p38-MAPK in PBMCs.

### Native SLY activates NF-κB

**Figure 7:**
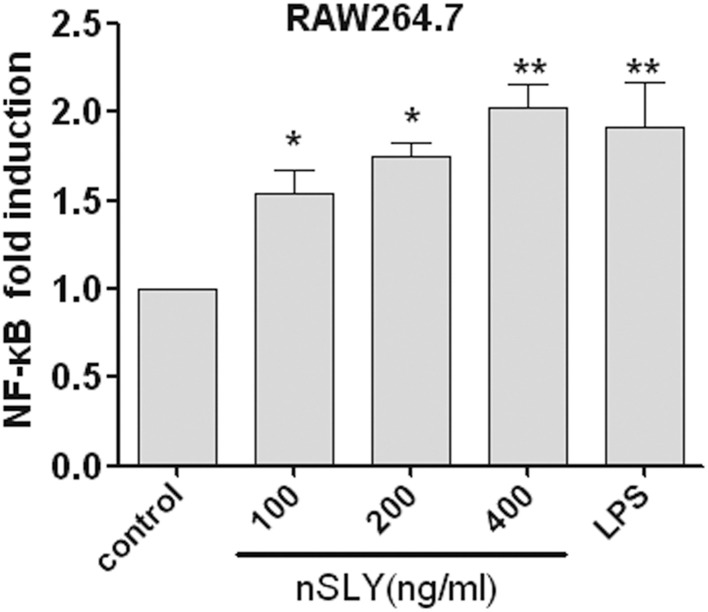
**Native SLY can activate NF-κB in RAW264.7**. RAW264.7 cells were transiently transfected with SV40-luciferase and NF-κB renilla-luciferase. Twelve hours after transfection, the cells were stimulated with LPS (100 ng/mL) or SLY (100–800 ng/mL) for 12 h. The cells were lysed, and the luciferase and renilla-luciferase acitivities were measured. Results were normalized for renilla-luciferase activity and represented as fold stimulation over the non-stimulated controls. The results are expressed as the mean ± SD for three experiments, and a One-Way ANOVA test was used for statistical analysis. ^*^P < 0.05; ^**^P < 0.01.

NF-κB (p50/p65) and AP-1 (c-Fos/c-Jun) are the important transcription factors of the TLR4-MAPK signal pathways activated by LPS, and they coordinate the induction of many genes encoding inflammatory mediators (Guha and Mackman, 2001). SLY induced TNF-α release from macrophages via TLR4 (Figures 4B–D). Therefore, the association of NF-κB and AP-1 activation with SLY-induced inflammation was evaluated in our study. Luciferase reporter experiments were performed to study the role of SLY in activating the NF-κB transcription factor. As shown in Figure 7, nSLY, similar to the positive control LPS, activated NF-κB in RAW264.7 cells; however, it did not activate AP-1 (data not shown). These results suggested that NF-κB might be involved in SLY-induced inflammatory activity.

## Discussion

The streptococcal toxic shock like syndrome (STSLS) in the large *S. suis* outbreak in 2005 in China caused a high incidence of morbidity and mortality despite antibiotic therapy (Sriskandan and Slater, 2006). The ST-7 strains that caused this outbreak have been reported to induce higher level of cytokine release in experimentally infected mice than ST-1 and ST-25. Moreover, serum samples from the 2005 outbreak revealed cytokine levels that were significantly higher in patients with STSLS than in patients with meningitis only. Researchers believe that ST7 has the ability to stimulate the production of massive amounts of proinflammatory cytokines, leading to STSLS (Ye et al., 2009).

Although some components of *S. suis*, including CPS, cell walls, and SLY have been reported to be inflammatory activators of endothelial cells (Vadeboncoeur et al., 2003) or immune cells (Lun et al., 2003; Graveline et al., 2007), the specific contributions of SLY to host inflammatory responses remain unclear. In this study, by comparing the inflammatory activity of WT *S. suis* and its isogenic *sly* mutant, we found that SLY is an important contributor to these responses. Interestingly, the ST-7 strain showed higher levels of SLY than the non-epidemic strains in our previous study (He et al., 2014) or in a study by another investigator (Ye et al., 2006). Therefore, the mechanisms underlying SLY induced inflammatory responses in hosts were studied. The results will possibly lead to effective rules to control inflammation and STSLS caused by *S. suis*.

In some studies, the concentrations of stimuli that induced inflammation were very high (purified CPS at 100 μg/ml and purified cell walls at 150 μg/ml), and the release of TNF-α (Graveline et al., 2007; Tanabe et al., 2010) was lower than in our study. In our study, a small amount of SLY (Å Mol/L) induced strong, long-term TNF-α release from human PBMCs (Figures 2B,C). The bacteria can easily be obtained from patient blood samples in China (Huong et al., 2014), and it is very dangerous when the bacteria enters the blood because Å mol/L of SLY can activate human PBMCs, resulting in the release large amounts of TNF-α throughout the body. We therefore used human PBMCs as an important immune cell type for study. Our results indicated that human PBMCs are more sensitive to SLY than THP-1 macrophages, RAW264.7 and primary peritoneal macrophages from mice. Further research should focus on how SLY interacts with human PBMCs. We demonstrated for the first time that SLY activates human PBMCs via TLR4. Moreover, SB203580, a p38-specific inhibitor, can completely inhibit TNF-α release from PBMCs, but not from THP-1. This means that p38 is a possible target to control TNF-α release induced by *S. suis* in host blood and that the specific inhibitors of p38 are potential therapeutic drugs. Host inflammation is a double-edged sword during host-pathogen interactions. It can help the host clear bacteria but can cause damage to the host when the inflammation is uncontrolled.

We found that the inflammatory activity of nSLY for PBMCs is not due to its haemolytic activity. The rSLY^P353V^ protein has less inflammatory activity than rSLY, but it demonstrates some activity with increasing stimulatory concentrations. This result was in contrast to the recent report that a vaccine candidate, the rSLY^P353L^ mutant, induces significantly less proinflammatory response than its wild-type counterpart (Du et al., 2013). This discrepancy might be due to the different models used in our study and the previous report. Although 60 μg of SLY and rSLY^P353L^ was used in a mouse model in that report, it is not clear that this amount of SLY is released during in the real infection process.

**Figure 8:**
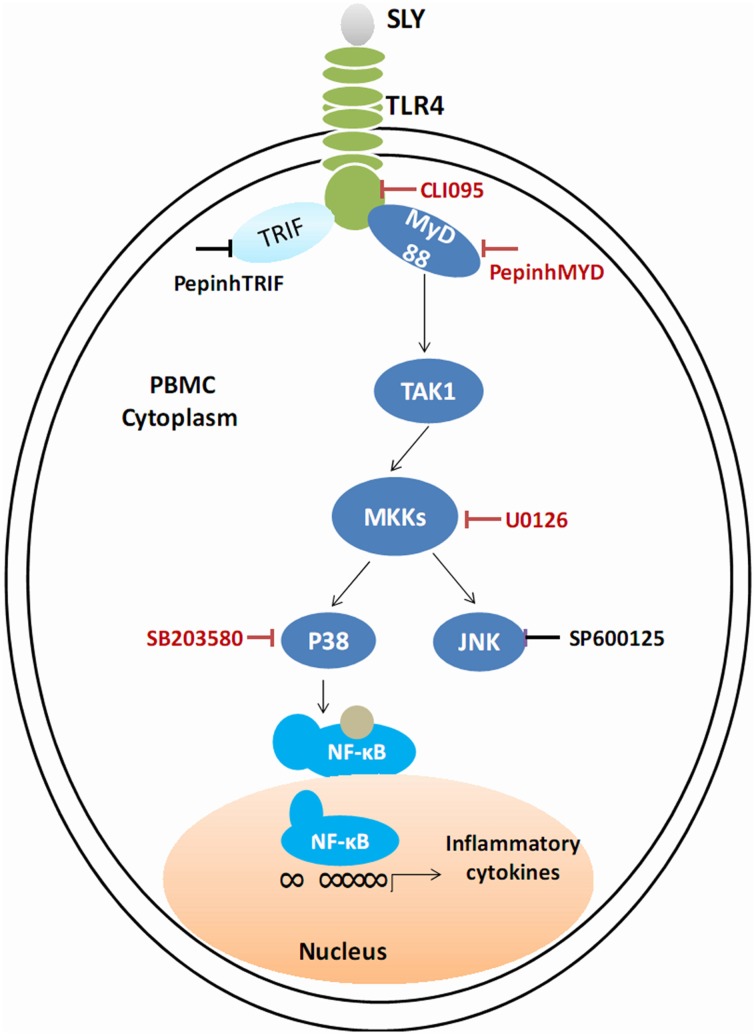
**Schematic of the cell signal pathway involved in the SLY-induced inflammatory response in PBMCs**. The TLR4-MyD88-p38MAPK signal transduction pathway in the SLY-induced inflammatory response in PBMCs is shown. The main effective inhibitors of this signal pathway are marked in red.

We conclude that TLR4 mediates innate immune responses to SLY, a CDC, independent of its cytolytic activity. SLY appears sensitive and powerful to human PBMCs. Given these results, we suggest that STSLS may be due to the robust inflammatory response to *S. suis*. This response is mediated by a TLR4-MyD88-p38 MAPK signal transduction pathway. In blood, SLY may be the main stimulus of the cytokine storm. Based on our results, the main effective inhibitor of the TLR4-MyD88-p38 MAPK signal transduction pathway in the SLY-induced inflammatory cytokine release is shown in Figure 8. From this, it may be possible to provide rules to treat STSLS caused by *S. suis* 2.

### Conflict of interest statement

The authors declare that the research was conducted in the absence of any commercial or financial relationships that could be construed as a potential conflict of interest.

## Supplementary material

Figure S1 **SLY is the main** ***S. suis*** **protein that stimulates of inflammation**. The gene expression of proinflammatory cytokines (IL-6, IL-8) in hCMEC/D3 cells induced by *S. suis* WT and the mutant strains (MOI = 10:1, stimulated for 2 h). Data are expressed as the mean ± SD of the fold increase of mRNA levels in the PBS group with SD for three independent experiments.

Figure S2 **The haemolytic activity of nSLY and the inhibiting effect of cholesterol**. The 2% erythrocytes were incubated with nSLY (10 μg/ml) pretreated with 0.1% β-ME or with nSLY (10 μg/ml) pretreated with 0.1% β-ME and cholesterol (1.5 μg/ml).
