# Supplementary material for: Toll-like receptor 4 confers inflammatory response to Suilysin
Source: Front Microbiol. 2015 Jun 26;6:644. doi: 10.3389/fmicb.2015.00644 (PMC4481166; doi:10.3389/fmicb.2015.00644)
Supplement: Supplementary file 1 [file Table1.DOCX]

**Supplemental information**

**Table S1. Primers Used in Real-time PCR analysis.**

| Gene | forward primer (5′- 3′) | reverse primer (5′- 3′) |
| --- | --- | --- |
| TNF-α IL-1β  IL-6  β-actin | TATGAGCCCATCTATCTG AATGACCTGAGCACCTTCT  ACCTCAGATTGTTGTTGT  CGAGAAGATGACCCAGAT | AATGATCCCAAAGTAGAC GCACATAAGCCTCGTTATCC  GTCCTAACGCTCATACTT  GATAGCACAGCCTGGATA |
