# Supplementary figures and images for: Toll-like receptor 4 confers inflammatory response to Suilysin
Source: Front Microbiol. 2015 Jun 26;6:644. doi: 10.3389/fmicb.2015.00644 (PMC4481166; doi:10.3389/fmicb.2015.00644)

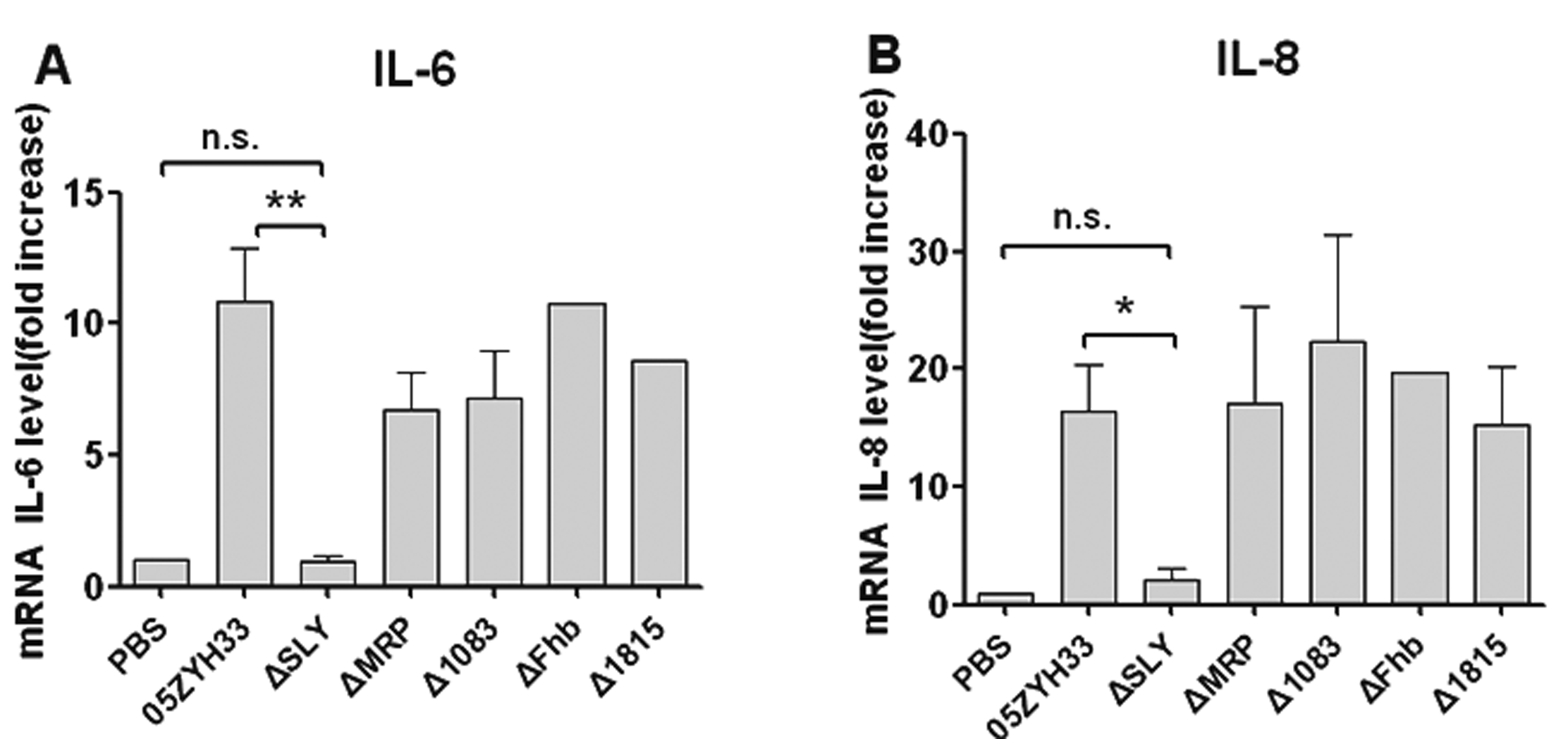

Supplement: Figure S1 — SLY is the main S. suis protein that stimulates of inflammation. The gene expression of proinflammatory cytokines (IL-6, IL-8) in hCMEC/D3 cells induced by S. suis WT and the mutant strains (MOI = 10:1, stimulated for 2 h). Data are expressed as the mean ± SD of the fold increase of mRNA levels in the PBS group with SD for three independent experiments. [file Image1.TIF]

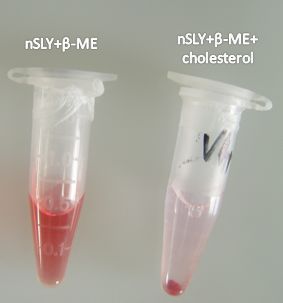

Supplement: Figure S2 — The haemolytic activity of nSLY and the inhibiting effect of cholesterol. The 2% erythrocytes were incubated with nSLY (10 μg/ml) pretreated with 0.1% β-ME or with nSLY (10 μg/ml) pretreated with 0.1% β-ME and cholesterol (1.5 μg/ml). [file Image2.JPEG]
